# The Influence of Angle Alpha, Angle Kappa, and Optical Aberrations on Visual Outcomes after the Implantation of a High-Addition Trifocal IOL

**DOI:** 10.3390/jcm11030896

**Published:** 2022-02-08

**Authors:** Guadalupe Cervantes-Coste, André Tapia, Claudia Corredor-Ortega, Mariana Osorio, Rafael Valdez, Martha Massaro, Cecilio Velasco-Barona, Roberto Gonzalez-Salinas

**Affiliations:** Anterior Segment Surgery Department, Asociación para Evitar la Ceguera en México I.A.P., Mexico City 04030, Mexico; andre.tapia.v@gmail.com (A.T.); corredorortegaclaudia@gmail.com (C.C.-O.); draoslom@gmail.com (M.O.); rvaldezsosa@gmail.com (R.V.); magamassaro@gmail.com (M.M.); ceciliovelascobarona@gmail.com (C.V.-B.); dr.gonzalezsalinas@gmail.com (R.G.-S.)

**Keywords:** multifocal IOL, MIOL, angle kappa, angle alpha, higher-order aberrations, defocus curve, visual outcome, correlation, patient selection

## Abstract

The aim of our investigation was to examine the possible correlations between optical aberrations, angle kappa, angle alpha, and visual outcomes following cataract surgery. In total, 56 eyes of 28 patients were implanted with the Liberty 677MY trifocal intraocular lens (IOL). Pre- and postoperative higher-order aberrations, coma, astigmatism, angle alpha, and angle kappa were registered, along with uncorrected and corrected visual acuities at multiple distances. Visual acuity and contrast sensitivity defocus curves were plotted, and the areas under the curve were calculated 1 and 3 months postoperatively. Excellent visual outcomes were found at all distances. Patients reported low levels of dysphotopsia, and 96.4% of patients achieved complete spectacle independence. While angle kappa significantly decreased during cataract surgery (*p* = 0.0007), angle alpha remained unchanged (*p* = 0.5158). Angle alpha correlated with postoperative HOAs and had a negative impact on near vision (*p* = 0.0543). Preoperative corneal HOA and coma had a strong adverse effect on future intermediate and near vision. Residual astigmatism significantly affected postoperative intermediate vision (*p* = 0.0091). Our results suggest that angle kappa is not an optimal predictive factor for future visual outcomes, while angle alpha and the preoperative screening of optical aberrations might help patient selection prior to multifocal IOL implantation.

## 1. Introduction

With the growth of people’s demand for high living standards, the interest in achieving long-term visual comfort and spectacle independence has increased [1]. The rapid development in cataract and refractive surgery has brought the ever-growing popularity of multifocal IOLs (MIOLs), which successfully respond to these needs, providing functional vision at multiple distances [2,3]. Although spectacle-free vision after surgery has become achievable, postoperative visual outcomes do not always meet the preoperative expectations [2,4]. The leading causes of dissatisfaction reported by the patients are ametropia, dysphotopic sensations, and reduced contrast sensitivity [1,5,6,7,8].

According to our current knowledge, not all patients will equally benefit from the implantation of MIOLs; therefore, careful patient selection and the identification of the factors characterizing the “good MIOL candidate” are of utmost importance [4,9]. Aside from conventional biometric parameters such as axial length, keratometry values, and anterior chamber depth, additional anatomic variables should be probably considered for inclusion in preoperative assessment and surgical planning [2,7,9].

**Figure 1:**
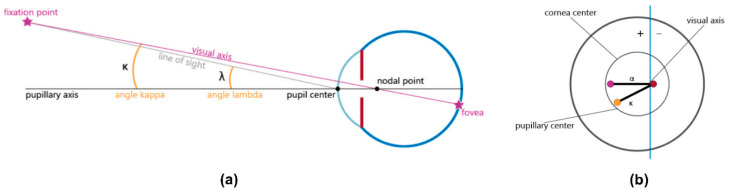
(**a**) Diagram of angle kappa (κ) formed by the visual axis and the pupillary axis; (**b**) graphic representation of angle kappa, visual axis, and pupillary axis, showing the center of the visual axis (red dot, representing the center of the reflection points), corneal center (violet dot in the diagram, similar to the anatomic center), and pupillary center (yellow dot, representing the center of the circle). The radial distance between the red dot and the violet dot represents angle alpha (α). The radial distance between the yellow dot and the red dot represents angle kappa (κ). The + sign represents the positive angle; the—sign represents the negative angle.

Pupil size, corneal and total astigmatism, coma, and other ocular aberrations are all reported to have impacts on the postoperative success [2,4,10]. Additionally, MIOLs are widely known to be sensitive to tilt and decentration [11,12,13,14,15,16,17,18]. Tilt and decentration are reported to affect the eye’s optical performance and, consequently, decrease optical quality [11,12,13,14,15,16,17,18]. These IOL dislocations might be correlated to the value of angle kappa (the angular distance between the visual and pupillary axis; Figure 1), which may also increase the risk of photic phenomena after MIOL implantation [11,13,19,20]. Similarly, angle alpha (the difference between the visual axis and the center of the limbus) may lead to poor centration of the MIOL, which can impair the postoperative visual performance, as well as elevate higher-order aberrations and ametropia in the pseudophakic eye [7,9,21,22].

Although there has been an increasing interest in clarifying the possible predictive value of optical aberrations, angle kappa, and angle alpha in patient selection prior to MIOL implantation, only limited scientific evidence is available. The published results are sometimes contradictory or provide only moderately strong evidence [1,2,7,9,11,12,13,19,20,23,24]. Additionally, the definition of angle kappa and alpha are not always the same in the literature, and measurement techniques often vary in different studies [25]. As a consequence, the published results are difficult to compare, and drawing appropriate conclusions is challenging.

The aim of our current investigation was to examine the visual outcomes following the implantation of a high-addition trifocal IOL and test whether there are any correlations between the actually achieved visual performance and the pre- and postoperative values of higher-order aberrations, coma, angle kappa, and angle alpha.

## 2. Materials and Methods

The current study was conducted according to the guidelines of the Declaration of Helsinki [26] and approved by COFEPRIS, Mexico City, Mexico (17 Cl 09 003 142; August 2019).

Prior to any intervention, each patient was informed in detail about the aim, process, and possible risks of both the surgery and our clinical investigation. Each of them gave their written consent on participating in the study.

Only adult subjects with age-related cataracts were enrolled in the patient group. None of the patients had preoperative corneal astigmatism of more than 1.5 diopters, and all of them had normal ocular health conditions apart from cataracts. Special care was taken not to include any subjects with congenital eye diseases, previous ocular trauma or ocular surgeries, or retinal disorders. None of the participants showed any signs of ocular inflammation, glaucoma, amblyopia, zonular instability, or any corneal diseases.

Bilateral cataract surgery with the standard stop-and-chop phacoemulsification technique was performed under topical anesthesia in each subject. The average interval between the first surgery and the surgery of the fellow eye was one week. All surgeries were performed by the same surgeon (G.C.-C.) between March 2019 and December 2020. Main clear corneal incisions of 2.2 mm on the steep meridian and a 1 mm secondary incision were employed for all surgeries. The DiscoVisc Ophthalmic Viscosurgical Device (OVD) (4% sodium chondroitin sulfate, 1.65% sodium hyaluronate) was applied during all surgeries (Alcon Laboratories, Fort Worth, TX, USA). After creating capsulorhexis manually, the cataractous lens was removed with cortical aspiration. A hydrophilic acrylic, trifocal presbyopia-correcting IOL with +1.75 diopters (D) intermediate and +3.5 D near addition (Liberty 677MY; Medicontur Medical Engineering Ltd., Zsámbék, Hungary) was implanted into the capsular bag in each case. The optimal spherical power of each lens was selected based on IOL calculations using the Haigis formula in the manufacturer’s online calculator (https://toriccalculator.net (accessed on 14 March 2019)). Target refraction was emmetropia in each case, and the personal surgical induced astigmatism (SIA) of the surgeon was taken into account in each calculation. The haptics of the IOL was positioned at 6 and 12 o’clock in each case. After removing all remaining OVD, the wounds were closed with 10.0 nylon sutures.

Preoperative measurements included a comprehensive examination of the anterior and posterior ocular segments including the retina. Apart from intraocular pressure (IOP), anterior and total keratometry values (K1, K2), axial length (AXL), corneal astigmatism, and subjective spherical and cylindric refractions (SPH, CYL, respectively) were registered. Uncorrected and corrected distance (UDVA, CDVA) and uncorrected intermediate (UIVA) and near (UNVA) visual acuities were measured. Photopic and mesopic pupillometry, the determination of total, corneal, and internal higher-order aberrations (HOAs), astigmatism, coma, and the measurement of angle kappa and angle alpha were performed with the OPD-Scan III wavefront aberrometer (Nidek Co., Ltd., Gamagori, Aichi, Japan). The refractive aberrations were expressed in root-mean-square (RMS) values.

The same measurements were repeated one month postoperatively. All patients were followed up for an additional visit three months following surgery. Subjective spherical and cylindric refraction, uncorrected and corrected distance, and uncorrected intermediate and near visual acuities were registered in each case. Patients were also asked whether they perceived any dysphotopic event (glare, halo, starburst, ghosting), and if they did, then how frequently they experienced them (never, seldom, from time to time, often, always). Additionally, they were asked if they had any further difficulties related to their vision. Possible adverse events or ocular comorbidities were examined and recorded in each case.

Monocular uncorrected and corrected visual acuity and contrast sensitivity defocus curves (VADC and CSDC, respectively) were measured and plotted one and three months following IOL implantation, using the Multifocal Lens Analyzer 3.0 application designed for iPad devices (Qvision, Madrid, Spain; defocuscurve.com (accessed on 2 January 2022)) [27]. During the measurements and analysis, the recommendations and protocols provided by the developers were applied [28]. The range of defocus was measured between +1.00 and −4.00 D with 0.5 D increments in each case. For each defocus curve, the system calculated the area under the curve (AUC) in the total range and in the far, intermediate, and near regions. A logMAR 0.3 value was used as the baseline of visual acuity AUC calculations.

The complete dataset obtained during the study is publicly available after de-identification in the Mendeley depository database from https://doi.org/10.17632/w5ws4pzzxr.1 (accessed on 6 December 2021) [29].

Statistical analysis was performed using the GraphPad Prism ver. 9.2.0 software (GraphPad Software Inc., San Diego, CA, USA). Descriptive statistics (mean, standard deviation, minimum, maximum, median, 95% confidence intervals, etc.) were calculated for each variable. The normality of each variable was tested using the D’Agostino and Pearson tests. The comparison of matching pre-and postoperative variables was tested using either the paired *t*-test or the nonparametric Wilcoxon test, based on the normal distribution of the data. Correlation analyses and linear regression analyses were performed to reveal the possible correlations between pre- and postoperative biometry and corneal characteristics and the visual outcomes. The partial correlation Spearman rho test was performed with the IBM SPSS Statistics 28.0 statistical software (IBM Co., Armonk, NY, USA). A *p*-value of <0.05 was considered statistically significant in each case. Data are presented as mean ± standard deviation (SD) in each case.

## 3. Results

Th pre- and postoperative data of 56 eyes of 28 subjects were included in the analysis. The preoperative characteristics of the patient population are summarized in Table 1. The mean age of the study group including 17 females (60.7%) and 11 males (39.3%) was 66.9 ± 10.1 years (min: 45; max 82 years).

### 3.1. Refractive and Visual Outcomes

Both spherical refraction and the spherical equivalent improved significantly following surgery (Preop. vs. Month 1: SPH: *p* = 0.110; SEQ: *p* = 0.0074; Preop vs. Month 3: SPH: *p* = 0.0053; SEQ: *p* = 0.0142), and the refractive outcomes were stable during the first three postoperative months (SPH: *p* = 0.8495; SEQ: *p* = 0.3339) (Table 2).

UDVA, CDVA, as well as UIVA and UNVA, showed significant improvement (*p* < 0.0001 in each case) (Table 2). The visual outcomes were stable; however, UDVA showed a further improvement between the first and third postoperative months (*p* = 0.0076).

**Figure 2:**
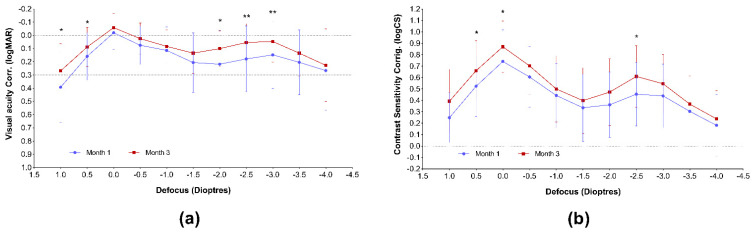
Monocular (**a**) corrected visual acuity and (**b**) contrast sensitivity defocus curves (mean ± SD), plotted 1 and 3 months postoperatively. Asterisks represent the significant statistical difference between the matching defocus values of the two curves. * *p* < 0.05; ** *p* < 0.01.

The corrected visual acuity and contrast sensitivity defocus curves are presented in Figure 2. Both curves represent sharp vision throughout the entire defocus range. The curves obtained at the second postoperative visit seem to be improved, compared with the month 1 results; however, a significant improvement could be revealed only between −2.0 and −3.0 diopters of defocus (50 to 33 cm distance from the eye) in the case of visual acuity, and only in the far range and at −2.5 D (40 cm) in the case of contrast sensitivity curve.

The area under the curve evaluation confirmed the improvement of near visual acuity between the first and third postoperative months (Table 3) and also proved a larger area in the total examined range.

Similarly, the AUC calculations affirmed the increase in the total and far range in the case of contrast sensitivity (Table 4).

### 3.2. Visual Quality—Spectacle Independence, Dysphotopsia, and Adverse Events

Nearly all 28 patients (96.4%) achieved spectacle independence at all distances. There was only one patient who occasionally required additional correction for one eye.

IOL decentration could be observed in 2 of 56 eyes (3.57%).

Dysphotopsia (halo, glare at night) was reported by 2 of the 28 patients (7.14%); however, their condition improved, and they rarely had any complaints by the end of the third postoperative month. The same two patients developed posterior capsule opacification (PCO), although they already showed posterior capsule metaplasia prior to cataract surgery. PCO could be detected in one eye of one additional patient. Nd:YAG laser capsulotomy was required in two eyes of two patients, between the 8th and 10th postoperative weeks.

### 3.3. Refractive Aberrations and Notable Angles (Kappa and Alpha)

Corneal, internal and total astigmatism, coma, higher-order aberrations, and kappa and alpha angles were determined before and one month following IOL implantation. The matching pre- and postoperative values were compared pairwise, and it was found that total coma and internal HOA were significantly reduced, compared with the preoperative levels (Table 5; *p* = 0.0259 and *p* = 0.0129, respectively). Similarly, angle kappa showed a significant decrease, both in photopic and mesopic conditions (*p* = 0.0007 and *p* < 0.0001, respectively). All other examined parameters, including angle alpha, were unchanged.

Correlation analyses were performed, to reveal the possible impact of preoperative angle kappa and angle alpha on future visual outcomes and on the amount of higher-order aberrations. Similarly, the potential contribution of preoperative HOAs and coma to the postoperative visual acuity was also evaluated. Furthermore, the impact of residual corneal astigmatism on visual outcomes was assessed.

No significant correlation could be found between the value of preoperative angle kappa and postoperative HOAs or visual acuities (Table 6). On the contrary, a weak correlation was revealed between the postoperative angle kappa and the actually achieved uncorrected intermediate vision (UIVA; *p* = 0.0219; Table 6).

Similarly, no correlations were found between preoperative angle alpha and the HOAs and visual acuities measured after surgery (Table 7). On the contrary, postoperative angle alpha was shown to have negative impact on intermediate vision (Table 7; r = −0.269; R^2^ = 0.098; *p* = 0.0219). No significant contribution to uncorrected far and near vision could be verified, although a higher number of cases would possibly reveal an existing correlation with angle alpha.

The possible impact of preoperative HOAs (total and corneal) and coma (total and corneal) on the postsurgical visual outcomes was also assessed (Table 8). Total preoperative HOA did not show any remarkable correlation with the future visual acuities, while corneal HOA was significantly correlated with postoperative CDVA (r = 0.417; R^2^ = 0.264; *p* = 0.0126), and UIVA (r = −0.459; R^2^ = 0.217; *p* = 0.0056). Total preoperative coma was found to have a remarkable negative impact on postoperative UIVA (r = −0.379; R^2^ = 0.042; *p* = 0.0296), and UNVA (r = −0.433; R^2^ = 0.104; *p* = 0.0118). Corneal preoperative coma had a significant influence on CDVA (r = 0.352; R^2^ = 0.210; *p* = 0.0411) and a large negative impact on UIVA (r = −0.536; R^2^ = 0.255; *p* = 0.0011).

Residual corneal astigmatism was found to have the largest impact on UIVA (Table 9; r = 0.365; R^2^ = 0.173; *p* = 0.0091), but no significant correlation could be found with either UDVA, CDVA, or UNVA.

## 4. Discussion

Implantation of multifocal IOLs offers a popular and efficient treatment for presbyopia correction during cataract surgery or refractive lens exchange; however, in some cases, visual outcomes do not meet the preliminary expectations [30]. Hence, the improvement of preoperative assessment and the identification of optical parameters that are predictive for future surgical outcomes are of high importance, as such measures can efficiently aid proper patient selection.

In the current study, we investigated the possible correlations between pre- and postoperative optical aberrations and visual outcomes following the binocular implantation of a high-addition MIOL.

UDVA, UIVA, UNVA, and CDVA all showed a significant improvement, compared with the preoperative values (<0.0001 in each case). Visual acuities measured 1 and 3 months postoperatively were similar, although a slight improvement of distance vision could be observed. This might be due to the modification of the ocular inner geometry after IOL implantation [31], neuroadaptation processes [32], or a slight decentration of the IOL, which was previously reported on the Liberty 677MY lens [17]. An important limitation of our current study is that exact measurements of IOL decentration were not performed, and therefore, its contribution to visual outcomes could not be assessed. Nevertheless, our visual acuity results are in good agreement with the measurements reported by other papers, following the implantation of the same MIOL [17,33].

The MLA iPad application proved to be a quick and easy-to-use tool in our clinical practice for obtaining both visual acuity results and contrast sensitivity defocus curves. Both curves confirm good quality vision along all of the defocus range, which is further confirmed by the complete spectacle independence achieved by the majority of the patients. Our VADCs are similar to those reported in previous studies after the implantation of the same presbyopia-correcting IOL [17,28]. We found it important to plot the visual acuity, in addition to contrast sensitivity defocus curves, as these curves are more sensitive to small changes in optical quality than VA [28,34]. Clinical studies evaluating differences in CS between MIOLs with clinical contrast sensitivity function (CSF) tests usually measure only the far distance, and their usefulness in detecting small differences in optical quality among MIOLs is questionable [28]. The measurement of CSDC is rather similar to the through focus response (TFR) in optical bench [28]. Furthermore, the letters used in the application are not comparable to sinusoidal gratings [28], and there is also a difference in background luminance between the two types of tests [28]. Hence, the CS measured with the MLA application is different from that usually measured with the conventional CSF technique, and comparing our results to other published data might be misleading. Nevertheless, we indeed believe that the CSDC provides a high, additional value by giving information on the visual quality throughout the entire range of vision and not only in the far distance region. The course of our CSDC curves corresponds to those reported by Fernández et al., who followed the implantation of the same MIOL, using the same iPAD application [35]. Nevertheless, the calculated AUCs are difficult to compare with those previously published by the Spanish group [17,28], as the exact defocus range and the definition of the far, intermediate, and near ranges slightly differ from that used in our evaluation. If we compare the AUCs calculated 1 and 3 months postoperatively, a significant improvement of near visual acuity can be observed (*p* = 0.0435). This led to an increase in the total VA-AUC, as well (*p* = 0.0324). Contrast sensitivity AUCs showed a remarkable advance in the far range (*p* = 0.0027), which consequently resulted in the growth of the total AUC (*p* = 0.0060). We suspect that, similarly to the conventional visual acuity results we had, these early changes after surgery are connected to the anatomical changes in the ocular inner geometry and neuroadaptation processes [31,32], although further investigations need to clarify the exact mechanisms behind.

**Figure 3:**
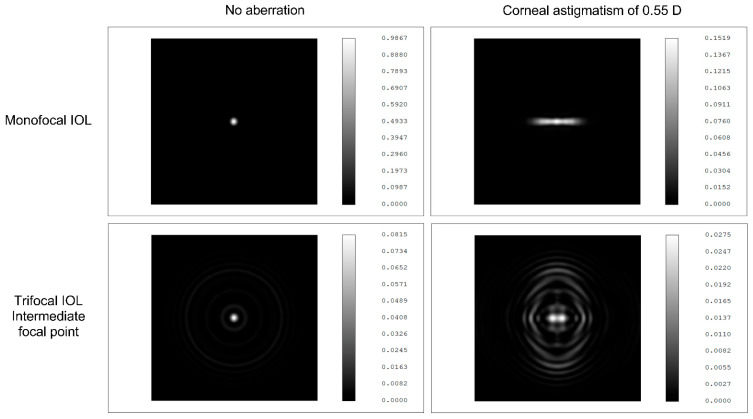
The effect of corneal astigmatism on the point spread function of a monofocal IOL and on the intermediate point spread function of a trifocal IOL. Simulations are created in an anatomical eye model with a 3.0 mm pupil diameter and monochromatic light (550 nm wavelength). Courtesy of Medicontur Medical Engineering Ltd.; unpublished measurements.

The matching pre- and postoperative values of the total, corneal, and internal optical aberrations were compared. Astigmatism did not change significantly, as mainly patients requiring no astigmatism correction were enrolled into the study group. Residual corneal astigmatism was, however, shown to positively correlate with the postoperative intermediate visual acuity (UIVA; *p* = 0.0091). Nochez et al. reported that ocular astigmatism created a clear vision zone between the first principal meridian and second principal meridian, hence increasing depth-of-focus values [36]. Nio et al. found that optical aberrations can increase the depth of focus while decreasing visual quality under optimum focus conditions [37]. Therefore, these aberrations play essential roles in the balance between acuity and depth of focus, the latter an especially important parameter in pseudophakic eyes [37]. In our current study, we could confirm the previous findings: astigmatism negatively correlated with contrast sensitivity [38]. These clinical observations are further supported by the simulations created in an anatomical eye model with a 3.0 mm pupil diameter and monochromatic light at 550 nm wavelength (Figure 3; unpublished data; courtesy of Medicontur Medical Engineering Ltd.). Uncorrected corneal astigmatism contributes to higher point spread function, more light scattering, and reduced image quality with monofocal IOLs (Bi-Flex 677ABY), compared with the setting without any aberration; but its negative impact on visual quality is remarkably larger in the intermediate range of the MIOL (Liberty 677MY) made from the same material and with the same design.

A significant decrease in total coma values could be observed (*p* = 0.0259). Similar findings were reported by other investigators, following the implantation of aspheric IOLs [7,39,40,41]. Increased coma values are believed to result in intolerable dysphotopsia, especially following the implantation of MIOLs with diffractive optics [4]. In our cohort, none of the patients complained of severe dysphotopsia, which might be in accordance with low coma RMS values. According to Santhiago et al., coma aberrations provide information on whether the IOL is properly centered [8]. Coma values were also hypothesized to be associated with IOL tilt [12,14]. In the current study, no IOL tilt could be detected in any of the eyes examined. A slight IOL dislocation was observed in one case, but the postoperative coma values were low (0.18), although somewhat higher than the preoperative value of the same eye (0.10). Based on this single event, drawing conclusions would be rather unfounded. It must be noted, however, that the preoperative total coma values were found to negatively correlate with the future intermediate and near visual outcomes (UIVA, UNVA). Additionally, corneal coma seemed to predict impaired postoperative UIVA, as well as CDVA. No previous data from the literature could be found to help understand this exact correlation. The etiology of these interactions remains to be elucidated by further examinations.

Neither total nor corneal HOAs showed remarkable differences between the pre- and postoperative values (*p* = 0.0815; *p* = 0.7493, respectively). In contrast, internal HOA showed a statistically significant decrease, compared with preoperative values. Similar results were published in a recent paper by Lee et al. [7], although they revealed a significant decrease in the internal and total HOAs. This might be due to the higher number of eyes (*n* = 73) included in their analysis, compared with the 56 eyes of our research. A Taiwanese investigation reported a correlation between corneal HOA and postoperative CDVA [7]. These findings were confirmed by our present research (r = 0.417; R^2^ = 0.264; *p* = 0.0126). Furthermore, we could also identify an existing inverse correlation between preoperative corneal HOA and UIVA. To prevent any confounding, we checked whether this correlation was not a false positive finding reflecting the possible impact of the residual SEQ. The partial correlation Spearman rho test (controlling for residual SEQ) confirmed that the association between the preoperative corneal HOA and postoperative UIVA was significant (r = −0.461; *p* = 0.006). As Maeda reported [42], a mild increase in HOAs can be the cause of suboptimal results with the multifocal IOLs. Furthermore, he proposed that the preoperative evaluation of corneal irregular astigmatism and the informed consent about the effects of corneal irregular astigmatism on quality of vision would be useful for avoiding the claims after surgery even for the candidates of conventional IOLs [42].

Kappa angle has been hypothesized to contribute to postoperative dysphotopic phenomena (halo and glare) following MIOL implantation [1,11,19,20]. Some authors also suggested using preoperative angle kappa as a predictor of future visual quality and assumed angle kappa to be used as a deciding factor in patient selection [11,19,20,24]. According to recent publications [2] and our own research, this might have been a premature conclusion. We found a significant difference between the pre- and postoperative angle kappa angles of the same eye, both in photopic and mesopic conditions (*p* = 0.0007 and *p* < 0.0001, respectively). Furthermore, preoperative angle kappa did not show any correlation with the visual acuities or HOA values measured postoperatively. This is in accordance with the recent findings after the implantation of various MIOLs (AT LISA tri 839MP, PanOptix) [1,2]. Large angle kappa was defined as the value that exceeds half of the diameter of the central optic zone of the MIOL [43]. According to the previous findings [44], this considerable angle kappa results in a greater chance of IOL decentration, which is likely to induce dysphotopsia and decrease visual quality [11,13]. In our current investigation, only two patients with posterior capsule opacification reported dysphotopsia, although both of them already showed posterior capsule metaplasia prior to cataract surgery. Their kappa angle sizes were not remarkably large, and no IOL dislocation could be observed in any of their eyes. Correlation analysis between angle kappa and visual acuity or contrast sensitivity defocus curve AUCs could not confirm any interaction either (data not shown). Our findings agree with those previously reported by Velasco-Barona et al. [2], and those published by Fernández et al.—namely, the kappa angle or chord mu did not have any relationship with AUCs [18]. This suggests that preoperative angle kappa may not be appropriate to predict future visual outcomes. Postoperative angle kappa, however, showed a negative correlation with UIVA (r = −0.334, R^2^ = 0.098, *p* = 0.0219), although the exact interpretation and clinical significance of this correlation require further investigation.

Unlike angle kappa, the size of angle alpha is related to the limbus, which is constant under every condition [9]. In our research, the size of angle alpha was confirmed to be unchanged during cataract surgery in both photopic and mesopic light conditions (*p* = 0.5158 and *p* = 0.4775, respectively). Multiple studies ascertained that angle alpha might yield a significant estimation value for postoperative vision, and it might be a predictive factor of image quality by MIOL patients [3,7,9,45]. Our results support the former findings: The postoperative angle alpha was found to have a negative impact on near vision (UNVA; r = −0.315, R^2^ = 0.113, *p* = 0.0543). According to previous studies, larger angle alpha may lead to poor IOL centration, and decentration might impair postoperative visual performance [7]. In contrast, Fernández et al. reported that a slight decentration of a low-addition trifocal lens may even be recommendable to achieve better visual acuity at near [18]. As we did not measure exact decentration in our study, this discrepancy is still to be resolved by further research. Furthermore, our data suggest that postoperative angle alpha is significantly associated with corneal and internal HOA (*p* = 0.0009 and *p* = 0.0291, respectively). Based on these results, we speculate that the association with the total HOA should also be significant (*p* = 0.0779), and a higher number of cases would likely confirm this assumption. Similarly, no statistically significant correlation between the preoperative angle alpha and postoperative HOAs could be detected. Despite this result, keeping in mind that the value of angle alpha is constant during cataract surgery, and considering the trend found in our correlation analyses (total HOA *p* = 0.0770; corneal HOA *p* = 0.0643), a larger dataset could presumably approve an existing correlation.

Our investigation has several limitations. We could only enroll only 56 eyes of 28 patients into our evaluation; however, a higher number of cases might have been beneficial to approve our initial findings. Furthermore, the range of patient age is large, and it might have had an impact on the results; however, the exact issues revolving around this topic were not among the purposes of our current research. A high proportion of cataract patients are diagnosed with preoperative corneal astigmatism [46,47,48], but since the toric model of the study lens (Liberty 677MTY) was not available in our country during the study period, several patients could not be included in our research. Additionally, the SARS-CoV−2 pandemic also hindered patient recruitment. Due to the limited number of subjects enrolled in the investigation, we decided to analyze the results of both eyes, although these might be correlated and might represent a possible bias during the evaluation. We are also aware that visual acuity and contrast sensitivity are subjective metrics of human vision, and visual perception is the joint performance outcome of the optical system of the eye and the neural mechanisms of the visual cortex. Although our results suggest that some optical aberrations contribute to the final visual outcomes, the exact correlations could only be evaluated with an objective, quality assessment of the retinal image. Strehl ratio, modular transfer function (MTF) cutoff, and objective scatter index (OSI) were, however, not recorded during our research. It should not be ignored either that brain activity is likely to be able to correct the possible impaired quality of retinal images deriving from optical aberrations [49]. Despite this fact, from a clinical perspective, the surgeons’ major aim is to restore the patients’ subjective vision, and therefore, the identification of preoperative predictors of future visual outcomes defined by subjective parameters may be acceptable and justified.

## 5. Conclusions

Based on our findings, angle kappa does not seem to be a reliable tool in patient selection prior to MIOL implantation, particularly as its significant decrease can be expected during surgery. It seems, however, that angle alpha, which is practically maintained during IOL insertion, has a much higher impact on future visual outcomes, defined as visual acuity and optical aberrations. Preoperative optical aberrations such as coma and HOA are likely to influence future visual outcomes as well, especially in the near and intermediate ranges. Further analyses with higher numbers of cases and appropriate interpretation are, however, crucial for a deeper understanding of the current topic.

## Figures and Tables

**Table 1:** Preoperative demographics and biometry values of the patient group.

Preoperative Parameters	Mean ± SD	Minimum	Maximum
Age	66.89 ± 10.1	45	82
K1 total keratometry (D)	43.21 ± 1.17	40.5	45.1
K1 anterior (D)	43.09 ± 1.18	39.75	45.2
K2 total keratometry (D)	43.78 ± 1.19	40.75	45.67
K2 anterior (D)	43.77 ± 1.25	40.75	45.75
AXL (mm)	23.68 ± 0.87	21.67	25.8
IOL power (D)	20.69 ± 2.71	13	26
Pupil photopic (mm)	3.03 ± 0.59	1.97	4.5
Pupil mesopic (mm)	4.96 ± 1.43	2.99	8.56

**Table 2:** Spherical and cylindric subjective refractions and visual acuities, measured preoperatively and 1 and 3 months following surgery.

Clinical Outcomes	Preoperative			Postoperative Month 1			Postoperative Month 3		
	Mean ± SD	Min	Max	Mean ± SD	Min	Max	Mean ± SD	Min	Max
SPH (D)	0.52 ± 2.58	−10.0	3.25	0.02 ± 0.46	−1.00	1.25	−0.01 ± 0.48	−1.25	1.25
CYL (D)	−0.54 ± 0.56	−2.00	0.25	−0.49 ± 0.44	−1.50	1.00	−0.27 ± 0.44	−1.25	1.00
SEQ (D)	0.25 ± 2.62	−10.0	3.13	−0.23 ± 0.47	−1.50	1.00	−0.15 ± 0.54	−1.25	1.50
Axis (degrees)	88.4 ± 49.4	5.00	180	79.6 ± 51.6	0.00	180	84.4 ± 54.2	0.00	180
UDVA (logMAR)	0.68 ± 0.53	0.18	2.4	0.12 ± 0.11	0.00	0.70	0.09 ± 0.09	0.00	0.40
CDVA (logMAR)	0.27 ± 0.41	0.00	2.4	0.04 ± 0.08	0.00	0.54	0.02 ± 0.05	0.00	0.18
UIVA (logMAR)	0.65 ± 0.16	0.30	0.88	0.17 ± 0.12	0.00	0.60	0.18 ± 0.14	0.00	0.40
UNVA (logMAR)	0.69 ± 0.16	0.30	0.88	0.12 ± 0.11	0.00	0.48	0.10 ± 0.09	0.00	0.30

**Table 3:** Visual acuity area under defocus curve measurements during the first three postoperative months.

Visual Acuity Area under Curve (AUC)	Mean ± SD	Minimum	Maximum	*p*-Value ^1^
Month 1 (*n* = 51)				
Total (+1.0 D to −3.5 D)	1.81 ± 0.98	0.00	4.35	0.0324
Far (+1.0 D to −0.5 D)	0.52 ± 0.20	0.00	0.90	0.0554
Intermediate (−0.5 D to −2.0 D)	0.53 ± 0.33	0.00	1.50	0.1987
Near (−2.0 D to −3.5 D)	0.65 ± 0.47	0.00	2.00	0.0435
Month 3 (*n* = 41)				
Total (+1.0 D to −3.5 D)	2.28 ± 0.91	0.23	3.92	
Far (+1.0 D to −0.5 D)	0.61 ± 0.20	0.09	0.92	
Intermediate (−0.5 D to −2.0 D)	0.64 ± 0.29	0.02	1.15	
Near (−2.0 D to −3.5 D)	0.87 ± 0.44	0.00	1.65	

^1^ *p*-values obtained with the paired *t*-test. *p* < 0.05 values were considered as statistically significant.

**Table 4:** Contrast sensitivity threshold area under defocus curve measurements during the first three postoperative months.

Contrast Sensitivity Threshold Area under Curve (AUC)	Mean ± SD	Minimum	Maximum	*p*-Value ^1^
Month 1 (*n* = 54)				
Total (+1.0 D to −3.5 D)	2.11 ± 1.64	0.00	6.35	0.0060
Far (+1.0 D to −0.5 D)	0.75 ± 0.44	0.00	1.73	0.0027
Intermediate (−0.5 D to −2.0 D)	0.57 ± 0.56	0.00	2.04	0.0635
Near (−2.0 D to −3.5 D)	0.63 ± 0.66	0.00	2.40	0.0725
Month 3 (*n* = 40)				
Total (+1.0 D to −3.5 D)	2.84 ± 1.76	0.12	7.19	
Far (+1.0 D to −0.5 D)	0.96 ± 0.43	0.12	1.78	
Intermediate (−0.5 D to −2.0 D)	0.74 ± 0.60	0.00	2.32	
Near (−2.0 D to −3.5 D)	0.89 ± 0.69	0.00	2.59	

^1^ *p*-values obtained with the paired *t*-test. *p* < 0.05 values were considered as statistically significant.

**Table 5:** Comparison of the pre- and postoperative values of the examined refractive aberrations and angle κ and angle α.

Optical Aberrations	Preoperative	Postoperative Month 1	*p*-Value ^1^
AST total (D)	−0.06 ± 0.78	−0.12 ± 0.77	0.6574
AST corneal (D)	−0.18 ± 0.85	−0.46 ± 0.81	0.5323
AST internal (D)	−0.31 ± 1.42	−0.15 ± 1.08	0.5683
Coma total (RMS)	0.32 ± 0.29	0.16 ± 0.34	0.0259
Coma corneal (RMS)	0.33 ± 0.45	0.16 ± 0.13	0.2766
Coma internal (RMS)	0.38 ± 0.52	0.25 ± 0.42	0.4661
HOA total (RMS)	0.97 ± 1.16	0.84 ± 3.25	0.0815
HOA corneal (RMS)	0.55 ± 0.69	0.40 ± 0.32	0.7493
HOA internal (RMS)	1.03 ± 1.32	0.91 ± 3.37	0.0129
Angle kappa photopic	0.35 ± 0.18	0.24 ± 0.11	0.0007
Angle kappa mesopic	0.40 ± 0.18	0.32 ± 0.12	<0.0001
Angle alfa photopic	0.56 ± 0.19	0.61 ± 0.29	0.5158
Angle alfa mesopic	0.63 ± 0.30	0.63 ± 0.36	0.4775

^1^ *p*-values obtained with the paired *t*-test or the Wilcoxon test, based on the normality of the data. *p* < 0.05 values were considered as statistically significant.

**Table 6:** Correlation between the pre- and postoperative angle kappa and the postoperative higher-order aberrations and visual outcomes (monocular visual acuities measured 1 month postoperatively).

Angle Kappa	R	95% C.I.	R^2^	*p*-Value ^1^
Preoperative				
HOA total (RMS)	−0.133	−0.50; 0.27	0.044	0.5172
HOA corneal (RMS)	0.036	−0.37; 0.42	0.000	0.8602
HOA internal (RMS)	0.082	−0.33; 0.47	0.002	0.6971
UDVA (logMAR)	−0.053	−0.41; 0.32	0.019	0.7758
CDVA (logMAR)	−0.128	−0.47; 0.24	0.029	0.4917
UIVA (logMAR)	−0.155	−0.49; 0.22	0.057	0.4043
UNVA (logMAR)	0.012	−0.35; 0.37	0.006	0.9475
Postoperative Month 1				
HOA total (RMS)	0.082	−0.21; 0.37	0.000	0.5857
HOA corneal (RMS)	0.204	−0.09; 0.47	0.014	0.1693
HOA internal (RMS)	0.206	−0.09; 0.48	0.068	0.1692
UDVA (logMAR)	−0.248	−0.51; 0.05	0.071	0.0925
CDVA (logMAR)	−0.187	−0.46; 0.12	0.045	0.2094
UIVA (logMAR)	−0.334	−0.57; −0.04	0.151	0.0219
UNVA (logMAR)	−0.269	−0.52; 0.03	0.098	0.0671

^1^ *p*-values obtained during the correlation analysis. *p* < 0.05 values were considered as statistically significant.

**Table 7:** Correlation between the pre- and postoperative angle alpha and the postoperative higher-order aberrations and visual outcomes (monocular visual acuities measured 1 month postoperatively).

Angle Alpha	R	95% C.I.	R^2^	*p*-Value ^1^
Preoperative				
HOA total (RMS)	−0.376	−0.69; 0.06	0.128	0.0770
HOA corneal (RMS)	−0.392	−0.70; 0.04	0.040	0.0643
HOA internal (RMS)	−0.204	−0.58; 0.25	0.025	0.3633
UDVA (logMAR)	0.150	−0.24; 0.50	0.031	0.4466
CDVA (logMAR)	0.112	−0.28; 0.47	0.010	0.5713
UIVA (logMAR)	−0.053	−0.43; 0.34	0.015	0.7893
UNVA (logMAR)	0.037	−0.35; 0.41	0.002	0.8526
Postoperative Month 1				
HOA total (RMS)	−0.290	−0.56; 0.04	0.076	0.0779
HOA corneal (RMS)	−0.517	−0.72; −0.23	0.206	0.0009
HOA internal (RMS)	−0.359	−0.62; −0.03	0.049	0.0291
UDVA (logMAR)	0.004	−0.32; 0.33	0.017	0.9795
CDVA (logMAR)	−0.305	−0.58; 0.03	0.070	0.0622
UIVA (logMAR)	−0.280	−0.56; 0.05	0.110	0.0881
UNVA (logMAR)	−0.315	−0.58; 0.02	0.113	0.0543

^1^ *p*-values obtained during the correlation analysis. *p* < 0.05 values were considered as statistically significant.

**Table 8:** Correlation between the preoperative aberrations (higher-order aberration and coma) and the future visual outcomes (monocular visual acuities measured 1 month postoperatively).

	R	95% C.I.	R^2^	*p*-Value ^1^
HOA total preop				
UDVA (logMAR)	−0.015	−0.37; 0.34	<0.001	0.9326
CDVA (logMAR)	0.226	−0.14; 0.54	0.002	0.2064
UIVA (logMAR)	−0.283	−0.58; 0.08	0.004	0.1101
UNVA (logMAR)	−0.245	−0.55; 0.12	0.097	0.1703
HOA corneal preop				
UDVA (logMAR)	0.083	−0.27; 0.41	0.026	0.6347
CDVA (logMAR)	0.417	0.09; 0.66	0.264	0.0126
UIVA (logMAR)	−0.459	−0.69; −0.14	0.217	0.0056
UNVA (logMAR)	−0.039	−0.38; 0.31	<0.001	0.8262
Coma total preop				
UDVA (logMAR)	−0.199	−0.52; 0.16	0.017	0.2653
CDVA (logMAR)	0.125	−0.24; 0.46	0.003	0.4898
UIVA (logMAR)	−0.379	−0.65; −0.03	0.042	0.0296
UNVA (logMAR)	−0.433	−0.68; −0.09	0.104	0.0118
Coma corneal preop				
UDVA (logMAR)	−0.030	−0.37; 0.32	0.001	0.8668
CDVA (logMAR)	0.352	0.01; 0.62	0.210	0.0411
UIVA (logMAR)	−0.536	−0.74; −0.23	0.255	0.0011
UNVA (logMAR)	−0.282	−0.57; 0.07	0.012	0.1063

^1^ *p*-values obtained during the correlation analysis. *p* < 0.05 values were considered as statistically significant.

**Table 9:** Correlation between the residual corneal astigmatism and the visual outcomes (monocular visual acuities measured 1 month postoperatively).

	R	95% C.I.	R^2^	*p*-Value ^1^
Residual Corneal Astigmatism				
UDVA (logMAR)	0.134	−0.16; 0.40	0.054	0.3524
CDVA (logMAR)	−0.085	−0.36; 0.21	<0.001	0.5558
UIVA (logMAR)	0.365	0.09; 0.59	0.173	0.0091
UNVA (logMAR)	0.255	−0.03; 0.50	0.068	0.0741

^1^ *p*-values obtained during the correlation analysis. *p* < 0.05 values were considered as statistically significant.

## Data Availability

The complete dataset obtained during the study is publicly available after de-identification in the Mendeley depository database from https://doi.org/10.17632/w5ws4pzzxr.1 (accessed on 6 December 2021) [29].
